# CPR Quality Assessment in Schoolchildren Training

**DOI:** 10.3390/jcdd9110398

**Published:** 2022-11-17

**Authors:** Katia M. G. Oliveira, Maria José C. Carmona, Antonio P. Mansur, Julio Y. Takada, Nino Fijačko, Federico Semeraro, Andrew Lockey, Bernd W. Böttiger, Naomi K. Nakagawa

**Affiliations:** 1 Kids Save Lives Brazil, Education, Assessment and Intervention in Cardiovascular and Pulmonary Group, Faculdade de Medicina, Universidade de São Paulo, Sao Paulo 05508-220, Brazil; 2 Anestesiology Discipline, Faculdade de Medicina, Universidade de São Paulo, Sao Paulo 05508-220, Brazil; 3 Serviço de Prevencao, Cardiopatia na Mulher e Reabilitação Cardiovascular, Instituto do Coracao (InCor), Hospital das Clinicas HCFMUSP, Faculdade de Medicina, Universidade de Sao Paulo, Sao Paulo 05403-900, Brazil; 4 Faculty of Health Science, University of Maribor, 2000 Maribor, Slovenia; 5 Anaesthesia and Intensive Care Department, Ospedale Maggiore, 40133 Bologna, Italy; 6 Calderdale and Huddersfield NHS Trust, Salterhebble, Halifax HX3 OPW, UK; 7 Department of Anaesthesiology and Intensive Care Medicine, University Hospital, Medical Faculty, University of Cologne, 50923 Cologne, Germany

**Keywords:** cardiopulmonary resuscitation, education, assessment, schoolchildren, basic life support

## Abstract

Whilst CPR training is widely recommended, quality of performance is infrequently explored. We evaluated whether a checklist can be an adequate tool for chest compression quality assessment in schoolchildren, compared with a real-time software. This observational study (March 2019–2020) included 104 schoolchildren with no previous CPR training (11–17 years old, 66 girls, 84 primary schoolchildren, 20 high schoolchildren). Simultaneous evaluations of CPR quality were performed using an observational checklist and real-time software. High-quality CPR was determined as a combination of 70% correct maneuvers in compression rate (100–120/min), depth (5–6 cm), and complete release, using a real-time software and three positive performance in skills using a checklist. We adjusted a multivariate logistic regression model for age, sex, and BMI. We found moderate to high agreement percentages in quality of CPR performance (rate: 68.3%, depth: 79.8%, and complete release: 91.3%) between a checklist and real-time software. Only 38.5% of schoolchildren (~14 years-old, ~54.4 kg, and ~22.1 kg/m^2^) showed high-quality CPR. High-quality CPR was more often performed by older schoolchildren (OR = 1.43, 95%IC:1.09–1.86), and sex was not an independent factor (OR = 1.26, 95%IC:0.52–3.07). For high-quality CPR in schoolchildren, a checklist showed moderate to high agreement with real-time software. Better performance was associated with age regardless of sex and BMI.

## 1. Introduction

Sudden cardiac arrest (SCA) is the third leading cause of death worldwide. Every year, approximately 700,000 deaths from SCA occur in North America and Europe [1,2,3]. Over 65% of SCA occurs at residences, and more than 200,000 deaths could be prevented by cardiopulmonary resuscitation (CPR) followed by medical care [4,5]. As prolonged hypoxia in critical tissues results in death, immediate high-quality CPR and the use of an automatic external defibrillator (AED) improves neurological outcomes and survival rates in out-of-hospital cardiac arrest (OHCA) [4,5,6,7,8,9,10,11,12]. In this context, several countries adopted measures to train laypeople in basic life support (BLS) [7,8,13,14]. In particular, young children and schoolchildren have been identified as a target population for CPR training, which has been advocated by the KIDS SAVE LIVES statement supported by the World Health Organization and the International Liaison Committee on Resuscitation [1,3,11,13,15,16].

High-quality CPR requires effective chest compressions (rate between 100–120/min, depth between 5–6 cm in adults, and complete chest release after compression) [11,12,17,18]. During training, the evaluation of CPR quality can be assessed by an observational checklist and also by real-time software [19,20,21,22]. The CPR quality evaluations performed by the two methods have previously showed moderate agreement in students over 17 years of age; however, studies evaluating schoolchildren’s performance are still lacking [19,20,21]. Therefore, this study aimed to evaluate whether an observational checklist can be an adequate tool for assessment of chest compression quality compared with a real-time software in schoolchildren. In addition, we examined the effect of different subject variables on performance of effective CPR.

## 2. Materials and Methods

This prospective longitudinal study was approved by the Ethical Committee of the University of São Paulo Medical School (CAAE: 05564819.1.0000.0065). We recruited students from a primary school and a secondary school in São Paulo City, between March 2019 and March 2020. Schoolchildren and their legal guardians gave the written informed assent and consent, respectively. Schoolchildren were 11 to 17 years old, of both sexes, and with no previous first aid or BLS training. Students that were not able to understand the BLS instructions or to perform BLS-related physical activities were excluded. Trained instructors and raters/assessors from the KIDS SAVE LIVES BRAZIL (KSLB) [15] project participated in this study.

### 2.1. Trainings and High-Quality CPR

CPR training was performed at the Skills Laboratory of the Medical School with three groups over two hours. Each group included one instructor and one assessor (for evaluation method) for five students using one adult torso manikin (Little Anne QCPR^®^, Laerdal Medical Inc., Stavanger, Norway) with real-time feedback software (QCPR training 4.13.3, Laerdal Medical Inc., Stavanger, Norway). The real-time software is considered the gold-standard tool for chest compression evaluation [8,10,17,18,20,22,23,24,25,26,27,28,29,30,31]. We determined high-quality CPR using a combination expressed as 70% correct maneuvers considering all the three skills of effective chest compression: rate (100 to 120/min), depth (50 to 60 mm), and complete release after compression, during a one-minute recording for each student [17,28,29,31,32,33]. KSLB raters/assessors completed a checklist by observational analysis of effective chest compressions that included (a) compression rate between 100–120/min, (b) compression depth between 5–6 cm, and (c) chest complete release after compression. The criteria rate was: yes = pass or no correct performance = fail, as performed by others [15,20,34]. We determined high-quality CPR using the checklist as the combination of three positive scorings (yes) in adequate chest compression rate, depth and release.

We also measured schoolchildren’s height and weight (MIC200PPA, Micheletti Ind., São Paulo, Brazil) to determine body mass index.

### 2.2. Statistical Analysis

We presented data of demographic characteristics and educational level as means and standard deviation or as numbers and percentages. We performed comparisons between “Effective” and “Ineffective” groups using T-test or Chi-Square test, depending on variables. Percentage of agreement between a checklist and real-time software were analyzed for each skill (compression rate, depth, and release). A multivariate logistic regression was used to assess the high-quality CPR (obtained by the real-time software) and adjusted by age, sex, and body mass index. A *p*-value < 0.05 was considered as significant.

## 3. Results

We included 110 students from two public schools as participants in this study. We excluded data from six students who were not able to achieve one minute of chest compression time recordings. From the remaining 104 subjects, 40 students (mean age of 13.9 years, BMI of 22.1 kg.m^−2^, 28 female) performed high-quality CPR that was measured by the real-time software. On the contrary, 64 students (mean age of 12.9 years, BMI of 21.6 kg.m^−2^, 38 female) failed to perform effective chest compression (Table 1).

We performed an analysis using the real-time software and the checklist (Table 2) to determine “Effective” (true positive) versus “Ineffective” (true negative) performances. We observed moderate to high agreement percentages of true positive and negative performances between the two methods in chest compression rate (68.3%), compression depth (79.8%), and chest release (91.3%) When we analyzed individual CPR skills, we observed false positives in chest compression (61%), depth (71%), and release (100%). We found disagreement in false negative in chest compression (0%), depth (5%), and release (3%).

We found effective compression rates in 50 students (mean age 13 years, *p* < 0.001) versus ineffective rates in 54 students (mean age 12 years), and compression depth in 80 students with higher body mass index (22.7 kg/m^2^, *p* < 0.001) versus 24 students with mean body mass index of 18.7 kg/m^2^. Therefore, we determined which factors were associated with the high-quality chest compression measured by real-time software. Age was an independent factor, and sex and BMI were not associated with high-quality chest compression (Table 3).

## 4. Discussion

Our study evaluated whether an observational checklist was an adequate tool for assessment of chest compression quality in schoolchildren undertaking CPR training, compared with real-time software. We showed moderate to high agreement percentages between both tools in chest compression rate (68.3%), depth (79.8%), and release (91.3%). High-quality chest compressions during CPR training were performed by only 38.5% of schoolchildren aged between 11 and 17 years. In addition, increased age was associated with high-quality CPR, regardless of sex and BMI in this study. 

All of the elements in the chain of survival in OHCA are important for the victim’s outcome. Training schoolchildren may improve bystander CPR rates and survival [3,11]. The European Resuscitation Council, American Heart Association, and other Councils emphasize high-quality chest compressions while waiting for the arrival of the emergency medical service or the use of the AED [11,12,18]. High-quality CPR has been proposed as a combination of correct performance in chest compressions considering chest compression rate, compression depth, and complete release by groups of investigators. The recommendations varied between studies from 50% to 80% for different ages and subject groups. For instance, 50% in students aged between 10–15 years, 60% in students aged between 17–18 years, and in medical students aged over 17 years, 70% in students aged between 8–12 years, and between 10–15 years, 75% in students aged between 16–18 years, and 80% in students aged between 9–16 years [17,19,20,21,22,29,31,33,35]. In our study, we used 70% as the setpoint of combined correct performances. We found that only 38.5% of schoolchildren (mean aged 13.9 ± 1.7 years) successfully performed high-quality CPR. Similarly, other investigators reported poor general performance in schoolchildren between 10–15 years, and they determined 13 years old as the minimum age to perform high-quality CPR [17].

We found two studies that compared these two methods, an observational checklist and real-time software, for evaluating CPR quality due to training in students over 17 years using combined performances in chest compression rate, depth, and release. Van Dawen and co-workers [20] showed that both tools were effective to assess CPR competences (setpoint as 60%) in medical students (mean age of 21.4 years) with moderate agreement between tools in compression rate (72.6%), depth (70.1%), and complete release (67.7%). Our study was performed in schoolchildren (11–17 years), and showed moderate to high agreements between the two evaluation methods considering compression rate (68.3%), depth (79.8%), and complete release (91.3%). We found more cases of agreement between the two methods when assessing effective skills. When evaluating poor performance in schoolchildren, we found a lower number of agreements, similar to the study with medical students [20]. It is worth noting that checklists are subjective tools for performance assessments [21]. The raters or assessors can easily identify correct performances while they may have more difficulties assessing incorrect chest compression maneuvers. Another aspect is that the observational checklist method seems to consistently overestimate student performance (effective skills with checklist and ineffective skills with real-time software assessment) in chest compression rate, depth and, release compared to the real-time software. This represents a high ‘false positive’ compression rate (effective with checklist and ineffective with real-time software) in the observational checklist scoring of effectiveness for the separate skills. 

Our study has limitations. There was a lower number of high school students (n = 20) compared with the number of the primary school students (n = 84). However, the sample size of high school children may not have affected our results, as older students are expected to demonstrate better skills for high-quality chest compression [2,8,28]. We identified age as an independent factor for high-quality chest compression (OR: 1.43). Some studies proposed physical characteristics such as weight higher than 50 Kg [8,17,23,24], height higher than 1.5 m [17,24,25,26,36], or body mass index exceeding 22 kg.m^−2^ [17,24,36] as variables that may directly affect effective chest compression, possibly because of the biomechanics of the CPR movements and minimum physical strength and endurance [17]. We reported in this study that body mass index was not associated with high-quality CPR (all three skills together). However, when we analyzed individual CPR skills, we observed deeper compression in students with higher body mass index, similarly to other studies [17,24,36]. Another aspect is that our study did not show any effect of sex on CPR quality, similar to others [8,23] that found no correlation of gender and fatigue on depth compression [25] and no differences between the age groups of 13, 14, and 15 years [32]. On the contrary, other studies showed deeper compressions performed by male schoolchildren [17,37,38]. Several variables that may be associated with these controversial results in schoolchildren with similar anthropometric characteristics can be raised. Among them, variability of hormone differences, and growth patterns [17]. Female students may also present anticipated puberty as well as they can also present higher level of engagement and motivation to attend CPR trainings and tasks [17,23,38]. 

To the best of our knowledge, this is the first study that shows the good utility of an observational checklist as a simple, easy, reliable, and low-cost method to evaluate CPR quality during schoolchildren’s training, with moderate to high agreement (>70%) with a real-time software considering chest compression rate, depth and release. High-quality CPR was performed by older schoolchildren, independent of sex and BMI.

## 5. Conclusions

In conclusion, a checklist showed moderate to high agreement with real-time software for high-quality CPR evaluations in schoolchildren. However, checklist may overestimate CPR skills of schoolchildren. Better performance was associated with age regardless of sex and BMI.

## Figures and Tables

**Table 1:** Demographic characteristics and educational level are presented as mean value and standard deviation or number of subjects and proportion between effective and ineffective groups.

	Ineffective Group n = 64	Effective Group n = 40	*p*-Value
**Age, years**	12.9 ± 1.6	13.9 ± 1.7	**0.003**
**Weight, kg**	52.6 ± 14.0	55.4 ± 13.5	0.316
**Height, cm**	1.55 ± 0.1	1.58 ± 0.1	0.088
**Body Mass Index, kg/m^2^**	21.6 ± 4.5	22.1 ± 4.5	0.622
**Sex, n (%)**			
**Male**	26 (68)	12 (32)	0.273 *^#^*
**Female**	38 (58)	28 (42)	
**Schooling level, n (%)**			
**Primary School**	57 (68)	27 (32)	**0.006** *^#^*
**Secondary School**	7 (35)	13 (65)	

^#^, Chi-Square Test.

**Table 2:** Analysis of agreement percentage for each compression skill between a checklist and a real-time software in the Effective vs. Ineffective groups of schoolchildren.

			Real-Time Software		Agreement Percentage
	**Checklist**		**Ineffective Group**	**Effective Group**	**%**
**Compression rate**			**n = 54**	**n = 50**	**68.3**
	Ineffective Group	**n = 21**	21 (39)	0 (0)	
	Effective Group	**n = 83**	33 (61)	50 (100)	
**Compression depth**			**n = 24**	**n = 80**	**79.8**
	Ineffective Group	**n = 11**	7 (29)	4 (5)	
	Effective Group	**n = 93**	17 (71)	76 (95)	
**Chest release**			**n = 6**	**n = 98**	**91.3**
	Ineffective Group	**n = 3**	0 (0)	3 (3)	
	Effective Group	**n =101**	6 (100)	95 (97)	

**Table 3:** Multivariate logistic regression to analyze high-quality chest compression as the dependent variable adjusted for age, sex and body mass index.

	OR (95% IC)	*p*-Value
**Age**	1.43 (1.09–1.86)	**0.008**
**Sex**	1.26 (0.52–3.07)	0.606
**BMI**	0.99 (0.90–1.09)	0.848

## Data Availability

The data presented in this study are available in this article.
